# Clinical characteristics and resource utilization of emergency department patients with obstructive sleep apnea

**DOI:** 10.1371/journal.pone.0326194

**Published:** 2025-06-17

**Authors:** Mengzhu Sun, Xingyu Zhang, Yan-Cun Liu, Jiao Pei, Hui Fan, Jianguo Guo

**Affiliations:** 1 The Affiliated Hospital of Qingdao University, Qingdao, Shandong, China; 2 Department of Communication Science and Disorders, School of Health and Rehabilitation Sciences, University of Pittsburgh, Pittsburgh, Pennsylvania, United States of America; 3 Department of Emergency Medicine, Tianjin Medical University General Hospital, Tianjin, China; 4 Sichuan Cancer Hospital & Institute, Sichuan Cancer Center, School of Medicine, University of Electronic Science and Technology of China, Chengdu, China; 5 Department of Epidemiology and Biostatistics, West China School of Public Health and West China Fourth Hospital, Sichuan University Chengdu, Chengdu, China; 6 Department of Preventive Medicine, North Sichuan Medical College, Nanchong, China; Xuzhou Central Hospital, The Xuzhou School of Clinical Medicine of Nanjing Medical University, CHINA

## Abstract

**Background:**

This study investigates the differences between patients with and without obstructive sleep apnea (OSA) in U.S. emergency departments (EDs), focusing on demographics, resource utilization, clinical characteristics, and outcomes.

**Methods:**

Using data from the 2016−2017 National Hospital Ambulatory Medical Care Survey Emergency Department Subfile (NHAMCS-ED), we analyzed adult ED visits. Patients were classified as having OSA based on documented diagnoses or ICD-10-CM codes. Outcomes included hospital and ICU admission rates, medical resource utilization (e.g., imaging, blood tests), and mortality. Logistic regression was used to adjust for confounders.

**Results:**

OSA accounted for approximately 5,985,955 (2.8%) annual ED visits. Compared to non-OSA patients, those with OSA were more likely to be male (adjusted OR: 1.34, 95% CI: 1.14–1.57) and older, with the highest prevalence in the 60–74 age group. OSA patients were more likely to visit for respiratory (16.4% vs. 10.1%) and cardiovascular symptoms (3.5% vs. 2.1%). They required higher levels of care, with elevated hospital (30.3% vs. 13.7%, adjusted OR: 1.27, 95% CI: 1.03–1.58). Resource use was significantly higher, including blood tests (75.0% vs. 54.9%, adjusted OR: 1.58, 95% CI: 1.26–1.98) and imaging (73.1% vs. 53.9%, adjusted OR: 1.30, 95% CI: 1.07–1.59).

**Conclusions:**

Patients with OSA represent a distinct subgroup in EDs, characterized by greater resource needs, higher acuity, and worse outcomes. These findings underscore the importance of optimizing care strategies for this population to reduce the clinical and economic burden.

## Background

Obstructive sleep apnea (OSA) is a prevalent and growing public health concern worldwide, with its incidence closely tied to rising obesity rates [1,2]. This sleep disorder is characterized by repetitive upper airway obstruction during sleep, leading to intermittent hypoxia, fragmented sleep, and significant physiological stress [3]. OSA is associated with various adverse health outcomes, including hypertension [4,5], diabetes [6,7], cardiovascular [8,9] disease, and increased risk of traffic accidents [10–12], which collectively contribute to substantial morbidity, mortality, and economic burden [13–15].

In the United States, OSA affects an estimated 2–4% of the general population, although the true prevalence is likely much higher due to underdiagnosis [16,17]. Despite its significant health implications, only a fraction of individuals with OSA receive appropriate diagnosis and treatment [18]. This gap in care not only exacerbates the disease burden but also places strain on healthcare systems, particularly emergency departments (EDs), which serve as critical access points for acute medical care.

Previous research has primarily focused on OSA’s associations with comorbid conditions, diagnostic strategies, and treatment interventions [1,3,4,6]. However, limited attention has been given to understanding the unique characteristics and healthcare needs of ED patients with OSA. Exploring these differences is essential for tailoring emergency care strategies, optimizing resource allocation, and improving patient outcomes.

This study aims to address this knowledge gap by analyzing the demographics, clinical characteristics, resource utilization, and outcomes of ED visits by patients with OSA compared to those without OSA. Using data from the nationally representative 2016–2017 National Hospital Ambulatory Medical Care Survey Emergency Department Subfile (NHAMCS-ED), we seek to determine whether OSA patients require distinct consideration in ED settings and to identify opportunities for improving their care pathways.

## Method

This study utilized data from the 2016–2017 NHAMCS-ED, a nationally representative survey conducted by the Centers for Disease Control and Prevention. The NHAMCS-ED employs a multistage, stratified sampling design to capture comprehensive information on emergency department (ED) visits from approximately 300 hospital-based EDs across the United States. The study population included all adult patients aged 18 years and older, comprising a total of 27,609 ED visits.

Patients were classified as having obstructive sleep apnea (OSA) if their ED records included an explicit OSA diagnosis or the International Classification of Disease, Tenth Revision, Clinical Modification (ICD-10-CM) code G47.3 (Sleep Apnea) in one of the provider diagnosis fields. The primary outcome of interest was the presence of an OSA diagnosis during the ED visit. Secondary outcomes included emergency severity, measured by the Emergency Severity Index (ESI), a five-level triage scale that ranks patient acuity from immediate (level 1) to non-urgent (level 5). Additional outcomes were hospital and intensive care unit (ICU) admission rates, resource utilization metrics (including blood tests, imaging such as X-rays and CT scans, and procedures like BiPAP/CPAP or intubation), and clinical outcomes such as mortality or leaving the ED without treatment.

To investigate these outcomes, the study examined a comprehensive set of covariates to adjust for potential confounding. These included demographic characteristics (age, sex, race/ethnicity, and geographic region), socioeconomic factors (insurance type and residence type), and visit-specific variables (day of the week, arrival by ambulance, seen within the past 72 hours, and reported pain level). Clinical indicators such as vital signs (temperature, heart rate, blood pressure), and the primary reason for the ED visit were also included. In addition, major comorbid conditions—including coronary artery disease, cerebrovascular disease, congestive heart failure, obesity, and diabetes—were incorporated into the adjusted models to better account for underlying health status.

Descriptive statistics were used to compare characteristics between patients with and without OSA, and chi-square tests were performed to assess differences in categorical variables. Logistic regression models were then constructed to evaluate the associations between OSA and secondary outcomes while adjusting for covariates. Adjusted odds ratios (ORs) with 95% confidence intervals (CIs) were calculated for each model to quantify the relationships. Missing data were handled using NHAMCS-ED’s sequential hot-deck imputation method for key variables, such as age and diagnosis codes, while median imputation was applied to other variables before running regression models. All statistical analyses were performed using SAS® software (Version 9.4), with a significance threshold set at a p-value of less than 0.05. To account for multiple comparisons, we applied the Benjamini-Hochberg procedure to control the false discovery rate (FDR) at 0.05. This method was selected to balance the control of Type I error with maintaining statistical power across multiple related outcome tests. P-values were adjusted accordingly, and outcomes that remained significant after FDR correction are indicated in the Results and tables.

The use of NHAMCS-ED’s robust design and statistical methods ensured the findings were nationally representative and accounted for potential confounding factors. This study utilized data from the National Hospital Ambulatory Medical Care Survey Emergency Department Subfile, a publicly available and fully anonymized dataset provided by the Centers for Disease Control and Prevention (CDC). As the data are de-identified and do not contain personally identifiable information, the study was exempt from ethics approval by the University of Pittsburgh Institutional Review Board under protocol STUDY24120115. No direct interaction with human participants occurred.

## Results

In 2016–2017, there were approximately 215,240,000 adult ED visits in the United States, averaging 107.62 million visits annually. Among these, 5,985,955 visits (2.8%) were made by patients with OSA, equating to roughly 2,992,978 visits per year. Patients with OSA demonstrated distinct demographic and clinical characteristics compared to those without OSA. According to Table 1, they were more likely to be male (49.3% vs. 42.6%) and older, with the highest prevalence observed in individuals aged 60–74 years (30.5%), followed by those aged 50–59 years (23.9%) and 75 years or older (17.9%). Geographically, the Midwest had the largest proportion of OSA patients (34.8%), while the lowest proportion was seen in the West (15.3%). Race and ethnicity distributions did not differ significantly between the OSA and non-OSA groups. However, OSA patients were more likely to arrive at the ED by ambulance (30.7% vs. 18.4%) and to report severe pain levels at presentation (39.2% vs. 36.4%). In addition, patients with OSA were more likely to have obesity (31.3% vs. 4.6%), diabetes (44.9% vs. 14.4%), and cardiovascular diseases compared to those without OSA.

**Table 1:** Baseline Characteristics of Patients Presenting to the ED, Stratified by OSA, NHAMCS 2016–2017.

	Unweighted Sample (%)			Weighted Sample (%)		
	All	NON-OSA	OSA	All	NON-OSA	OSA
	27,609	26,867(97.3)	742(2.7)	215,240,000	209,254,639(97.2)	5,985,955(2.8)**
**Male**	12,031(43.6)	11,659(43.4)	372(50.1)**	92,150,415(42.8)	89,200,810(42.6)	2,949,604(49.3)
**Age**			**			**
18–39	11,576(41.9)	11,485(42.7)	91(12.3)	90,164,918(41.9)	89,459,033(42.8)	705,886(11.8)
40–49	4,237(15.4)	4,114(15.3)	123(16.6)	32,430,413(15.1)	31,478,989(15.0)	951,425(15.9)
50–59	4,338(15.7)	4,169(15.5)	169(22.8)	33,595,252(15.6)	32,165,451(15.4)	1,429,802(23.9)
60–74	4,496(16.3)	4,261(15.9)	235(31.7)	34,864,200(16.2)	33,038,871(15.8)	1,825,329(30.5)
≥ 75	2,962(10.7)	2,838(10.6)	124(16.7)	24,185,810(11.2)	23,112,296(11.0)	1,073,514(17.9)
**Race/ethnicity**			**			
NH White	12,731(60.3)	12,324(60.0)	407(70.1)	103,482,638(61.0)	100,112,868(60.8)	3,369,770(69.4)
Hispanic White	1,550(7.3)	1,537(7.5)	13(2.2)	13,708,707(8.1)	13,624,289(8.3)	84,418(1.7)
NH Black	4,796(22.7)	4,682(22.8)	114(19.6)	39,425,837(23.3)	38,345,594(23.3)	1,080,242(22.3)
Hispanic Black	70(0.3)	70(0.3)	0(0.0)	535,701(0.3)	535,701(0.3)	0(0.0)
Hispanic(Other)	1,096(5.2)	1,073(5.2)	23(4.0)	7,622,180(4.5)	7,405,840(4.5)	216,340(4.5)
Asian	548(2.6)	537(2.6)	11(1.9)	3,114,715(1.8)	3,058,266(1.9)	56,449(1.2)
Other	325(1.5)	312(1.5)	13(2.2)	1,687,547(1.0)	1,641,612(1.0)	45,934(0.9)
**Residence type**			**			
Private residence	25,607(94.7)	24,923(94.8)	684(93.8)	199,456,518(95.0)	193,883,542(95.0)	5,572,976(94.1)
Nursing home	506(1.9)	480(1.8)	26(3.6)	4,326,842(2.1)	4,130,477(2.0)	196,365(3.3)
Homeless	485(1.8)	475(1.8)	10(1.4)	2,310,912(1.1)	2,268,434(1.1)	42,478(0.7)
Other	434(1.6)	425(1.6)	9(1.2)	3,907,893(1.9)	3,800,237(1.9)	107,656(1.8)
**Insurance type**			**			**
Private insurance	7,380(29.3)	7,211(29.4)	169(25.0)	54,212,897(28.1)	52,872,485(28.2)	1,340,412(24.4)
Medicare	6,499(25.8)	6,160(25.2)	339(50.1)	51,235,274(26.6)	48,406,667(25.9)	2,828,608(51.5)
Medicaid or CHIP	7,916(31.5)	7,771(31.7)	145(21.4)	58,963,047(30.6)	57,819,397(30.9)	1,143,651(20.8)
Uninsured	2,482(9.9)	2,470(10.1)	12(1.8)	20,699,501(10.7)	20,591,757(11.0)	107,744(2.0)
Other	889(3.5)	878(3.6)	11(1.6)	7,636,327(4.0)	7,567,818(4.0)	68,509(1.2)
**Year**			**			*
2016	15,012(54.4)	14,648(54.5)	364(49.1)	111,728,252(51.9)	108,942,082(52.1)	2,786,170(46.5)
2017	12,597(45.6)	12,219(45.5)	378(50.9)	103,512,342(48.1)	100,312,557(47.9)	3,199,785(53.5)
**Day of ED Visit**						
Sunday	3,561(12.9)	3,471(12.9)	90(12.1)	27,650,051(12.9)	26,767,614(12.8)	882,437(14.7)
Monday	4,506(16.3)	4,383(16.3)	123(16.6)	35,199,890(16.4)	34,243,351(16.4)	956,540(16.0)
Tuesday	4,120(14.9)	3,998(14.9)	122(16.4)	32,023,200(14.9)	31,086,093(14.9)	937,107(15.7)
Wednesday	3,997(14.5)	3,906(14.5)	91(12.3)	31,013,896(14.4)	30,239,107(14.5)	774,789(12.9)
Thursday	3,852(14.0)	3,742(13.9)	110(14.8)	30,387,602(14.1)	29,481,090(14.1)	906,512(15.1)
Friday	3,857(14.0)	3,748(14.0)	109(14.7)	29,846,966(13.9)	29,041,275(13.9)	805,691(13.5)
Saturday	3,716(13.5)	3,619(13.5)	97(13.1)	29,118,988(13.5)	28,396,109(13.6)	722,879(12.1)
**Arrive by ambulance**	5,074(18.9)	4,870(18.6)	204(28.3)**	39,310,535(18.7)	37,504,678(18.4)	1,805,856(30.7)**
**Seen within last 72 hours**	870(3.4)	850(3.4)	20(2.9)	6,121,292(3.1)	5,963,221(3.1)	158,071(2.9)
**Pain level at Presentation**						
No pain	4,831(24.8)	4,705(24.8)	126(24.1)	34,913,498(23.2)	33,971,276(23.2)	942,221(23.4)
Mild	1,868(9.6)	1,819(9.6)	49(9.4)	14,203,227(9.4)	13,854,709(9.4)	348,518(8.7)
Moderate	6,019(30.8)	5,865(30.9)	154(29.5)	46,610,296(30.9)	45,455,534(31.0)	1,154,762(28.7)
Severe	6,801(34.8)	6,608(34.8)	193(37.0)	54,927,003(36.5)	53,349,572(36.4)	1,577,431(39.2)
**Temperature at Presentation**						
>36 °C–38 °C	24,663(95.8)	24,014(95.8)	649(94.2)	192,666,320(95.8)	187,477,777(95.9)	5,188,543(94.7)
≤ 36 °C	731(2.8)	701(2.8)	30(4.4)	5,509,078(2.7)	5,275,632(2.7)	233,445(4.3)
>38 °C	363(1.4)	353(1.4)	10(1.5)	2,885,402(1.4)	2,825,648(1.4)	59,753(1.1)
**Heart Rate at Presentation**						
≤ 90	18,407(66.7)	17,899(66.6)	508(68.5)	142,798,596(66.3)	138,769,015(66.3)	4,029,580(67.3)
90–100	4,584(16.6)	4,462(16.6)	122(16.4)	35,256,649(16.4)	34,254,963(16.4)	1,001,686(16.7)
100–110	2,526(9.2)	2,463(9.2)	63(8.5)	20,014,593(9.3)	19,570,984(9.4)	443,608(7.4)
110–120	1,235(4.5)	1,206(4.5)	29(3.9)	10,048,447(4.7)	9,773,532(4.7)	274,916(4.6)
> 120	857(3.1)	837(3.1)	20(2.7)	7,122,309(3.3)	6,886,144(3.3)	236,165(3.9)
**DBP at Presentation**						*
60–80	12,221(44.3)	11,923(44.4)	298(40.2)	93,823,839(43.6)	91,571,346(43.8)	2,252,493(37.6)
< 60	2,725(9.9)	2,645(9.8)	80(10.8)	20,826,919(9.7)	20,054,363(9.6)	772,556(12.9)
> 80	12,663(45.9)	12,299(45.8)	364(49.1)	100,589,836(46.7)	97,628,930(46.7)	2,960,906(49.5)
**SBP at Presentation**			**			*
80–120	6,283(22.8)	6,166(23.0)	117(15.8)	47,481,290(22.1)	46,505,061(22.2)	976,229(16.3)
< 80	1,064(3.9)	1,041(3.9)	23(3.1)	7,939,792(3.7)	7,667,953(3.7)	271,839(4.5)
> 120	20,262(73.4)	19,660(73.2)	602(81.1)	159,819,512(74.3)	155,081,624(74.1)	4,737,888(79.2)
**Census Region**			**			**
Northeast	4,503(16.3)	4,364(16.2)	139(18.7)	33,427,214(15.5)	32,139,982(15.4)	1,287,233(21.5)
Midwest	6,756(24.5)	6,504(24.2)	252(34.0)	52,153,223(24.2)	50,068,544(23.9)	2,084,679(34.8)
South	9,720(35.2)	9,528(35.5)	192(25.9)	83,789,428(38.9)	82,090,477(39.2)	1,698,951(28.4)
West	6,630(24.0)	6,471(24.1)	159(21.4)	45,870,728(21.3)	44,955,636(21.5)	915,092(15.3)
Visit Related to Injury**	8,493(30.8)	8,304(30.9)	189(25.5)	65,644,383(30.5)	64,281,400(30.7)	1,362,984(22.8)
Coronary artery disease**	2,155(7.8)	1,965(7.3)	190(25.6)	16,997,002(7.9)	15,218,436(7.3)	1,778,566(29.7)
Cerebrovascular disease**	1,110(4.0)	1,036(3.9)	74(10.0)	8,955,097(4.2)	8,371,715(4.0)	583,382(9.7)
Congestive heart failure**	1,162(4.2)	1,031(3.8)	131(17.7)	9,290,403(4.3)	8,194,952(3.9)	1,095,451(18.3)
Obesity**	1,453(5.3)	1,219(4.5)	234(31.5)	11,420,112(5.3)	9,548,658(4.6)	1,871,454(31.3)
Diabetes**	4,030(14.6)	3,716(13.8)	314(42.3)	32,821,647(15.2)	30,131,587(14.4)	2,690,059(44.9)

*Note: the missing proportion for residency type and arriving by ambulance is less than 5%; for insurance, temperature and seen within 72h, 5% − 10%; for race/ethnicity, 20% − 25%; for pain level, 29%.*

Pearson’s Chi-squared test was performed on unweighted samples, and Rao-Scott corrected chi-squared test was performed on weighted samples.

*p < 0.05, **p < 0.01.

According to Table 2 and Supplement S1 Table, patients with OSA were more likely to present with specific symptoms compared to general symptoms. Respiratory symptoms were more common among OSA patients (16.4% vs. 10.1%), with an adjusted odds ratio (OR) of 1.25 (95% CI: 0.95–1.65). Similarly, cardiovascular and lymphatic symptoms were more frequently observed in OSA patients (3.5% vs. 2.1%), corresponding to an adjusted OR of 1.43 (95% CI: 0.93–2.21). These findings highlight the increased burden of respiratory and cardiovascular complications among OSA patients.

**Table 2:** Selected Reason for Visit and Emergency Department Diagnosis among ED Patients with OSA, NHAMCS 2016-2017.

Reason for ED Visit	Unweighted Sample (%)			Weighted Sample (%)		
	All	NON-OSA	OSA**	All	NON-OSA	OSA**
General	5,305(19.2)	5,154(19.2)	151(20.4)	41,926,806(19.5)	40,556,326(19.4)	1,370,480(22.9)
Psychiatric	1,146(4.2)	1,110(4.1)	36(4.9)	7,841,631(3.7)	7,651,012(3.7)	190,619(3.2)
Neurologic	2,031(7.4)	1,970(7.3)	61(8.2)	15,867,786(7.4)	15,402,142(7.4)	465,643(7.8)
Cardiovascular and Lymphatic	591(2.1)	561(2.1)	30(4.0)	4,601,441(2.1)	4,394,614(2.1)	206,827(3.5)
Eyes and/or Ears	563(2.0)	549(2.0)	14(1.9)	4,397,893(2.1)	4,331,282(2.1)	66,611(1.1)
Respiratory	2,732(9.9)	2,626(9.8)	106(14.3)	21,990,645(10.2)	21,010,869(10.1)	979,776(16.4)
Digestive	4,360(15.8)	4,264(15.9)	96(12.9)	35,521,482(16.5)	34,759,685(16.6)	761,798(12.7)
Genitourinary	1,490(5.4)	1,469(5.5)	21(2.8)	10,469,363(4.9)	10,287,145(4.9)	182,218(3.0)
Dermatologic	796(2.9)	780(2.9)	16(2.2)	6,390,594(3.0)	6,276,843(3.0)	113,751(1.9)
Musculoskeletal	4,073(14.8)	3,954(14.7)	119(16.0)	32,172,694(15.0)	31,174,906(14.9)	997,788(16.7)
Other	4,484(16.3)	4,392(16.4)	92(12.4)	33,769,883(15.7)	33,119,439(15.8)	650,444(10.9)

*Note: The missing proportion for reasons for visit is less than 2%.*

Pearson’s Chi-squared test was performed on unweighted samples, and Rao-Scott corrected chi-squared test was performed on weighted samples.

*p < 0.05, **p < 0.01.

According to Tables 3 and 4, the analysis also revealed significantly higher medical resource utilization among OSA patients. Blood tests were performed in 75.0% of OSA cases, compared to 54.9% of non-OSA cases (adjusted OR: 1.58, 95% CI: 1.26–1.98). Imaging studies were more common in the OSA group, with 73.1% undergoing any imaging compared to 53.9% in the non-OSA group (adjusted OR: 1.30, 95% CI: 1.07–1.59). X-rays were performed in 56.1% of OSA cases versus 36.7% of non-OSA cases (adjusted OR: 1.29, 95% CI: 1.07–1.56). CT scans were also slightly more frequent in the OSA group (27.4% vs. 21.4%), although the adjusted OR (0.95, 95% CI: 0.77–1.77) did not reach statistical significance. Clinical outcomes further highlighted the severity of illness among OSA patients. These patients were significantly more likely to be classified as having a higher acuity level, with 26.0% assigned an Emergency Severity Index (ESI) score of 1 or 2 (immediate or emergent) compared to 14.6% of non-OSA patients (adjusted OR: 1.35, 95% CI: 1.02–1.77). Hospital admission rates were markedly higher in the OSA group (30.3% vs. 13.7%), with an adjusted OR of 1.27 (95% CI: 1.03–1.58).

**Table 3:** Proportion of Emergency Severity Index, Hospital admission, ICU admission, medical resources utilization, stratified by OSA, NHAMCS 2016-2017.

	Unweighted Sample (%)			Weighted Sample (%)		
	All	NON-OSA	OSA	All	NON-OSA	OSA
ESI score			**			**
1 (Immediate)	189(1.0)	185(1.0)	4(0.7)	1,474,896(1.0)	1,445,316(1.0)	29,580(0.7)
2 (Emergent)	2,621(13.1)	2,488(12.8)	133(23.7)	21,345,538(14.0)	20,248,550(13.6)	1,096,988(25.3)
3 (Urgent)	10,134(50.8)	9,844(50.8)	290(51.7)	76,939,174(50.3)	74,764,448(50.3)	2,174,726(50.1)
4 (Semi-urgent)	6,046(30.3)	5,922(30.5)	124(22.1)	46,377,544(30.3)	45,432,249(30.6)	945,295(21.8)
5 (Non-urgent)	959(4.8)	949(4.9)	10(1.8)	6,877,151(4.5)	6,781,709(4.6)	95,443(2.2)
Hospital Admission	3,854(14.0)	3,642(13.6)	212(28.6)**	30,582,913(14.2)	28,769,043(13.7)	1,813,870(30.3)**
ICU	469(1.7)	438(1.6)	31(4.2)**	3,852,739(1.8)	3,596,358(1.7)	256,381(4.3)**
Death in ED or hospital	117(0.4)	114(0.4)	3(0.4)	971,853(0.5)	939,700(0.4)	32,153(0.5)
Left before/after triage	774(2.8)	766(2.9)	8(1.1)**	6,245,255(2.9)	6,192,043(3.0)	53,213(0.9)**
Blood test performed	15,082(54.6)	14,540(54.1)	542(73.0)**	119,303,863(55.4)	114,814,564(54.9)	4,489,299(75.0)**
Any imaging performed	14,496(52.5)	13,984(52.0)	512(69.0)**	117,181,932(54.4)	112,805,327(53.9)	4,376,606(73.1)**
X-ray in ED	9,805(35.5)	9,423(35.1)	382(51.5)**	80,087,700(37.2)	76,726,914(36.7)	3,360,786(56.1)**
CT in ED	5,737(20.8)	5,542(20.6)	195(26.3)**	46,480,608(21.6)	44,837,476(21.4)	1,643,131(27.4)**
Ultrasound in ED	1,653(6.0)	1,605(6.0)	48(6.5)	12,996,039(6.0)	12,630,246(6.0)	365,793(6.1)
MRI in ED	307(1.1)	298(1.1)	9(1.2)	2,607,475(1.2)	2,534,138(1.2)	73,338(1.2)
Other Imaging in ED	359(1.3)	350(1.3)	9(1.2)	2,394,747(1.1)	2,327,695(1.1)	67,052(1.1)
Procedure	13,448(48.7)	13,099(48.8)	349(47.0)	106,962,016(49.7)	104,188,206(49.8)	2,773,810(46.3)
Waiting time (mean [95% CI])	37.7(36.8-38.6)	37.7(36.8-38.6)	38.7(33.6-43.8)	37.8(36.9-38.7)	37.7(36.8-38.6)	41.1(34.5-47.8)

*Note: the missing proportion for waiting time is 15%, for ESI score is 28%. “Waiting time” refers to the time from arrival to seeing the physician.*

Pearson’s Chi-squared test was performed on unweighted samples, and Rao-Scott corrected Chi-squared test was performed on weighted samples.

*p < 0.05, **p < 0.01.

**Table 4:** Odds Ratio of Emergency Severity Index, Hospital Admission, ICU Admission, Medical Resources Utilization for Patients with versus without OSA, NHAMCS 2016–2017.

	Crude Odds Ratio	p value	Adjusted Odds Ratio	p value
ESI Score: 1 or 2 vs. 4 or 5	2.63(2.06-3.35)	<.0001	1.35(1.02-1.77)	0.0332
ESI Score: 3 vs. 4 or 5	1.51(1.23-1.86)	<.0001	1.03(0.84-1.28)	0.7554
Hospital Admission	2.55(2.17-3.00)	<.0001	1.27(1.03-1.58)	0.0269
ICU	2.63(1.81-3.82)	<.0001	1.17(0.75-1.83)	0.4938
Left	0.37(0.19-0.75)	0.0056	0.66(0.31-1.42)	0.286
Blood test	2.30(1.95-2.70)	<.0001	1.58(1.26-1.98)	<.0001
Any imaging	2.05(1.75-2.40)	<.0001	1.30(1.07-1.59)	0.0087
X-ray	1.96(1.70-2.27)	<.0001	1.29(1.07-1.56)	0.0085
CT	1.37(1.16-1.62)	0.0002	0.95(0.77-1.17)	0.6173
Ultrasound	1.09(0.81-1.47)	0.575	1.07(0.74-1.56)	0.7159
MRI	1.10(0.56-2.13)	0.7904	0.56(0.24-1.32)	0.1824
Procedure	0.92(0.79-1.06)	0.241	0.89(0.75-1.06)	0.193

*Note: Adjusted variables include gender, age group, race/ethnicity, residence type, insurance type, census region, year, day of the week, arrival by ambulance, seen within last 72 hours, pain level, temperature, heart rate, dialytic blood pressure, injury status, reason for visit, coronary artery disease, cerebrovascular disease, congestive heart failure, obesity, and diabetes.*

All p-values were adjusted using the Benjamini-Hochberg procedure to account for multiple comparisons (FDR threshold = 0.05). Outcomes remaining significant after adjustment included blood test use and any imaging. Hospital admission was marginally significant.

## Discussion

This study provides a comprehensive analysis of emergency department visits by patients with obstructive sleep apnea in the United States using nationally representative data from the NHAMCS-ED. Our findings reveal that while OSA accounts for a relatively small proportion of ED visits (2.8%), patients with OSA demonstrate distinct clinical characteristics, higher resource utilization, and significantly worse outcomes compared to patients without OSA. These results highlight the substantial burden OSA places on emergency care systems and underscore the need for improved management strategies for this population [19].

Patients with OSA in this study were more likely to present with respiratory or cardiovascular symptoms compared to general symptoms, consistent with the known associations of OSA with respiratory dysfunction and cardiovascular complications such as hypertension, arrhythmias, and heart failure. These findings align with previous research emphasizing the bidirectional relationship between OSA and chronic respiratory [20–23] and cardiovascular diseases [24–27]. For example, OSA-induced hypoxemia [28] and sympathetic overactivation [29] may exacerbate preexisting conditions, potentially explaining the higher acuity levels observed in our study. Furthermore, the increased prevalence of musculoskeletal symptoms among OSA patients warrants additional investigation, as these may reflect overlapping conditions like obesity-related joint pain or generalized fatigue and muscle weakness due to poor sleep quality.

From a healthcare resource perspective, our results indicate that ED patients with OSA utilize significantly more resources than their non-OSA counterparts. This includes nearly double the rate of blood tests and significantly higher rates of imaging, particularly X-rays and CT scans. The higher resource utilization observed in this group may reflect the complexity of managing OSA patients, who often present with multiple comorbidities requiring comprehensive evaluation [30]. These findings suggest that healthcare providers in ED settings should be prepared for the diagnostic and therapeutic challenges posed by OSA patients. The study also highlights stark differences in outcomes. Patients with OSA were significantly more likely to require hospital or ICU admission. These adverse outcomes underscore the severity of OSA-related health complications and the importance of early intervention and management.

Notably, the demographic analysis revealed that OSA disproportionately affects older adults and males, consistent with known epidemiological trends [31,32]. However, the lack of significant differences in OSA prevalence across racial and ethnic groups in this study contrasts with prior findings suggesting disparities in OSA diagnosis and treatment [33]. This discrepancy may reflect the limitations of administrative data or differences in how OSA is documented across populations.

Despite the robustness of our findings, this study has limitations. First, the identification of OSA relied on documented diagnoses or ICD-10-CM codes within the NHAMCS-ED dataset. The reliance on administrative data introduces the potential for misclassification or underdiagnosis of OSA [34], particularly for patients without a prior diagnosis who may not have been recognized as having the condition during their ED visit. In addition, information on OSA severity (e.g., mild, moderate, severe) and treatment status, such as adherence to positive airway pressure (PAP) therapy, was not available, limiting our ability to assess the impact of disease control on outcomes. Second, although we adjusted for important comorbidities including coronary artery disease, cerebrovascular disease, congestive heart failure, obesity, and diabetes in our regression models, the possibility of residual confounding cannot be completely ruled out. NHAMCS-ED lacks information on certain patient characteristics that may influence outcomes, such as smoking status, economic status, employment status, and detailed medication use. Third, while we included a variable indicating whether patients had visited the ED within the previous 72 hours, the dataset does not capture longer-term patterns of healthcare utilization, which could influence resource use and outcomes. Fourth, the NHAMCS-ED dataset captures only the index ED visit and immediate disposition outcomes (e.g., hospital admission, death during hospitalization) without long-term follow-up data. Thus, we were unable to assess outcomes beyond the ED stay or subsequent hospitalization. Finally, due to the cross-sectional design of NHAMCS-ED, causal relationships between OSA and healthcare utilization or outcomes cannot be inferred. Our findings reflect associations rather than causal effects. Future longitudinal studies are needed to better characterize the impact of OSA and its management on emergency care outcomes over time.

## Conclusion

This study provides critical insights into the unique characteristics, resource needs, and outcomes of ED patients with OSA. Compared to patients without OSA, those with OSA exhibit higher acuity, greater resource utilization, and higher rates of hospital. These findings underscore the importance of improving the recognition and management of OSA in emergency settings [35,36]. To address the challenges identified in this study, healthcare systems should consider implementing targeted strategies to optimize care for OSA patients [37–39]. Potential interventions for future research include the development of ED-based OSA screening protocols, such as the STOP-BANG questionnaire, to identify high-risk patients who may otherwise go undiagnosed. Integration of automated flagging systems within electronic health records (EHRs) for patients with a known diagnosis of OSA could assist ED providers in recognizing individuals at elevated risk for complications. In addition, the creation of targeted care pathways—such as expedited respiratory therapy evaluations, early initiation of non-invasive ventilation, or referral to outpatient sleep specialists—may improve both immediate and long-term outcomes. Future studies should evaluate the feasibility, effectiveness, and cost-efficiency of these interventions in ED environments. Broader efforts, such as multidisciplinary approaches to managing OSA comorbidities, enhanced provider education on OSA-related complications, and public health initiatives promoting early diagnosis and treatment in outpatient settings, could also reduce the burden of OSA on emergency care systems. Longitudinal research is needed to explore the long-term outcomes of ED patients with OSA and to assess the impact of targeted management strategies. By addressing these gaps, we can better support patients with OSA, alleviate their clinical and economic burden, and enhance the overall efficiency and quality of emergency care delivery.

## Supporting information

S1 Table Association Between OSA Status in ED Patients and Their Visiting Characteristics (NHAMCS 2016–2017). (DOCX)
