# Supplementary material for: Clinical characteristics and resource utilization of emergency department patients with obstructive sleep apnea
Source: PLoS One. 2025 Jun 17;20(6):e0326194. doi: 10.1371/journal.pone.0326194 (PMC12173181; doi:10.1371/journal.pone.0326194)
Supplement: S1 Table — (DOCX) [file pone.0326194.s001.docx]

Supplement Table 1. Association Between OSA Status in ED Patients and Their Visiting Characteristics (NHAMCS 2016–2017)

|  | | Crude OR (95% CI) | p value | | Adjusted OR (95% CI) | | p value |
| --- | --- | --- | --- | --- | --- | --- | --- |
| Age | |  |  | |  | |  |
| 18–39 | | Reference [1] |  | | Reference [1] | |  |
| 40–49 | | 3.77(2.87-4.96) | 0.9374 | | 2.60(1.95-3.45) | | <.0001 |
| 50–59 | | 5.12(3.96-6.62) | <.0001 | | 3.00(2.29-3.95) | | <.0001 |
| 60–74 | | 6.96(5.45-8.89) | <.0001 | | 3.08(2.32-4.07) | | <.0001 |
| ≥ 75 | | 5.51(4.19-7.25) | <.0001 | | 2.49(1.79-3.47) | | <.0001 |
| Male vs. Female | | 1.31(1.13-1.52) | 0.0003 | | 1.34(1.14-1.57) | | 0.0003 |
| Race/ethnicity | |  |  | |  | |  |
| NH White | | Reference [1] |  | | Reference [1] | |  |
| NH Black | | 0.74(0.60-0.91) | 0.8992 | | 0.88(0.71-1.11) | | 0.2792 |
| Hispanic | | 0.41(0.29-0.57) | 0.0001 | | 0.58(0.41-0.83) | | 0.003 |
| Asian | | 0.62(0.34-1.14) | 0.4586 | | 0.71(0.38-1.34) | | 0.2884 |
| Other | | 1.26(0.72-2.22) | 0.0281 | | 1.35(0.73-2.47) | | 0.3374 |
| Day of Week | |  |  | |  | |  |
| Sunday | | Reference [1] |  | | Reference [1] | |  |
| Monday | | 1.08(0.82-1.43) | 0.8092 | | 1.03(0.77-1.38) | | 0.8489 |
| Tuesday | | 1.18(0.89-1.55) | 0.2255 | | 1.16(0.87-1.56) | | 0.3066 |
| Wednesday | | 0.90(0.67-1.21) | 0.0888 | | 0.85(0.63-1.17) | | 0.3196 |
| Thursday | | 1.13(0.86-1.50) | 0.4553 | | 1.14(0.85-1.53) | | 0.3951 |
| Friday | | 1.12(0.85-1.49) | 0.532 | | 1.06(0.79-1.43) | | 0.7107 |
| Saturday | | 1.03(0.77-1.38) | 0.7901 | | 1.03(0.76-1.39) | | 0.8701 |
| Year | |  |  | |  | |  |
| 2016 | | Reference [1] |  | | Reference [1] | |  |
| 2017 | | 1.25(1.08-1.44) | 0.0033 | | 1.19(1.02-1.38) | | 0.0297 |
| Residence type | |  |  | |  | |  |
| Private residence | | Reference [1] |  | | Reference [1] | |  |
| Nursing home | | 1.97(1.32-2.95) | 0.0008 | | 1.02(0.65-1.58) | | 0.9492 |
| Homeless | | 0.77(0.41-1.44) | 0.2406 | | 0.95(0.49-1.85) | | 0.8897 |
| Other | | 0.77(0.40-1.50) | 0.2691 | | 0.65(0.32-1.30) | | 0.2199 |
| Insurance type | |  |  | |  | |  |
| Private insurance | | Reference [1] |  | | Reference [1] | |  |
| Medicare | | 2.35(1.95-2.83) | <.0001 | | 1.22(0.98-1.51) | | 0.0729 |
| Medicaid or CHIP | | 0.80(0.64-1.00) | 0.4264 | | 0.86(0.68-1.09) | | 0.2057 |
| Uninsured | | 0.21(0.12-0.37) | <.0001 | | 0.33(0.18-0.60) | | 0.0003 |
| Other | | 0.54(0.29-0.99) | 0.2141 | | 0.56(0.30-1.07) | | 0.078 |
| Temperature | |  |  | |  | |  |
| 36 °C–38 °C | | Reference [1] |  | | Reference [1] | |  |
| ≤ 36 °C | | 1.58(1.09-2.30) | 0.0771 | | 1.20(0.81-1.79) | | 0.369 |
| > 38 °C | | 1.05(0.56-1.98) | 0.5849 | | 1.03(0.53-2.03) | | 0.924 |
| Heart Rate | |  |  | |  | |  |
| ≤ 90 | | Reference [1] |  | | Reference [1] | |  |
| 90–100 | | 0.96(0.79-1.18) | 0.5501 | | 1.05(0.85-1.30) | | 0.6778 |
| 100–110 | | 0.90(0.69-1.18) | 0.9455 | | 1.00(0.75-1.32) | | 0.971 |
| 110–120 | | 0.85(0.58-1.24) | 0.6629 | | 0.86(0.57-1.29) | | 0.4723 |
| > 120 | | 0.84(0.54-1.32) | 0.6847 | | 0.84(0.52-1.36) | | 0.4801 |
| DBP | |  |  | |  | |  |
| 60–80 | | Reference [1] |  | | Reference [1] | |  |
| < 60 | | 1.21(0.94-1.55) | 0.3768 | | 0.94(0.72-1.23) | | 0.6368 |
| > 80 | | 1.18(1.01-1.38) | 0.3756 | | 1.09(0.92-1.29) | | 0.3047 |
| Pain level | |  |  | |  | |  |
| No pain | | Reference [1] |  | | Reference [1] | |  |
| Mild | | 1.01(0.72-1.41) | 0.9139 | | 1.26(0.88-1.80) | | 0.205 |
| Moderate | | 0.98(0.77-1.25) | 0.6214 | | 1.33(1.07-1.66) | | 0.0107 |
| Severe | | 1.09(0.87-1.37) | 0.3436 | | 1.57(1.22-2.03) | | 0.0006 |
| 72-h revisit vs. Not | | 1.20(0.77-1.89) | 0.4203 | | 0.87(0.55-1.39) | | 0.5692 |
| Arrival by Ambulance vs. Not | | 1.72(1.46-2.03) | <.0001 | | 1.15(0.95-1.39) | | 0.1502 |
| Census Region | |  |  | |  | |  |
| Northeast | | Reference [1] |  | | Reference [1] | |  |
| Midwest | | 1.22(0.99-1.50) | <.0001 | | 0.97(0.77-1.21) | | 0.78 |
| South | | 0.63(0.51-0.79) | <.0001 | | 0.52(0.41-0.66) | | <.0001 |
| West | | 0.77(0.61-0.97) | 0.059 | | 0.77(0.60-0.99) | | 0.0374 |
| Reason for Visit (by Symptom Module) |  | | |  | |  | |
| General | | Reference [1] |  | | Reference [1] | |  |
| Psychiatric | | 1.11(0.77-1.60) | 0.3044 | | 1.43(0.96-2.12) | | 0.0774 |
| Neurologic | | 1.06(0.78-1.43) | 0.3512 | | 1.23(0.90-1.69) | | 0.1989 |
| Cardiovascular and Lymphatic | | 1.83(1.22-2.73) | 0.0002 | | 1.43(0.93-2.21) | | 0.1023 |
| Eyes and/or Ears | | 0.87(0.50-1.52) | 0.7652 | | 1.53(0.86-2.71) | | 0.1451 |
| Respiratory | | 1.38(1.07-1.77) | 0.0002 | | 1.25(0.95-1.65) | | 0.1065 |
| Digestive | | 0.77(0.59-1.00) | 0.0609 | | 0.94(0.71-1.23) | | 0.6454 |
| Genitourinary | | 0.49(0.31-0.77) | 0.0015 | | 0.93(0.57-1.49) | | 0.7487 |
| Dermatologic | | 0.70(0.42-1.18) | 0.2116 | | 0.94(0.54-1.63) | | 0.8169 |
| Musculoskeletal | | 1.03(0.81-1.31) | 0.3547 | | 1.10(0.84-1.44) | | 0.473 |
| Other | | 0.72(0.55-0.93) | 0.012 | | 0.94(0.69-1.28) | | 0.6896 |
| Visit Related to Injury vs. Not | | 1.31(1.11-1.55) | 0.0016 | | 0.95(0.77-1.18) | | 0.6481 |
| Coronary artery disease vs. No | | 9.69(8.22-11.43) | <.0001 | | 6.60(5.50-7.93) | | <.0001 |
| Cerebrovascular disease vs. No | | 2.76(2.16-3.54) | <.0001 | | 1.22(0.93-1.62) | | 0.1537 |
| Congestive heart failure vs. No | | 5.37(4.41-6.56) | <.0001 | | 1.65(1.29-2.10) | | <.0001 |
| Obesity vs. No | | 4.36(3.68-5.18) | <.0001 | | 1.68(1.37-2.07) | | <.0001 |
| Diabetes vs. No | | 4.57(3.94-5.31) | <.0001 | | 2.10(1.77-2.49) | | <.0001 |

*Note*: the adjusted OR was from a logistic regression including all variables in the table.
